# The Earliest Report

**Published:** 1924-05

**Authors:** Frank G. Hazell

**Affiliations:** Manchester Royal Infirmary


### "The Earliest Report."

Sir,—1. The Income and Expenditure account is kept on the Uniform System of Hospital Accounts.

2. All purchases are treated as expenditure and are analysed direct from the cash payment.

3. Liabilities incurred but not paid are not recorded in the books.

4. Liabilities outstanding at the end of the year are not brought into account, neither are they included in the balance-sheet.

5. Items of income are not recorded in the books before receipt.

6. Amounts outstanding due from debtors at the end of the year are not brought into account, neither are they included in the balance-sheet as assets.

7. Separate records are not kept of the expenditure on in-patients and out-patients respectively.

8. The basis upon which the in-patient and out-patient statistics are prepared is as follows:—The cost of the out-patients' and casualty departments is ascertained as accurately as possible, and then deducted from the cost of maintenance and administration.

I have answered "Enquirer's" eight-point questionnaire as briefly as possible, but I will gladly answer more fully any point upon which he seeks further information with regard to the method of keeping the accounts at the Manchester Royal Infirmary.

Frank G. Hazell.

Manchester Royal Infirmary.

